# Supplementary material for: Healthcare provider perspective on barriers and facilitators in the care of pediatric injury patients at a tertiary hospital in Northern Tanzania: A qualitative study
Source: PLOS Glob Public Health. 2023 Nov 20;3(11):e0002599. doi: 10.1371/journal.pgph.0002599 (PMC10659160; doi:10.1371/journal.pgph.0002599)
Supplement: Attachment — Submitted filename: Response to Reviewers.docx [file pgph.0002599.s004.docx]

**Response to Reviewers:**

Journal Requirements:

**1. Please review your reference list to ensure that it is complete and correct. If you have cited papers that have been retracted, please include the rationale for doing so in the manuscript text, or remove these references and replace them with relevant current references. Any changes to the reference list should be mentioned in the rebuttal letter that accompanies your revised manuscript. If you need to cite a retracted article, indicate the article’s retracted status in the References list and also include a citation and full reference for the retraction notice.**

Thank you for this instruction. The reference list has been reviewed to ensure it is complete and correct. We are not citing any papers that have been retracted.

**2. Please include a complete copy of PLOS’ questionnaire on inclusivity in global research in your revised manuscript. Our policy for research in this area aims to improve transparency in the reporting of research performed outside of researchers’ own country or community. The policy applies to researchers who have travelled to a different country to conduct research, research with Indigenous populations or their lands, and research on cultural artefacts. The questionnaire can also be requested at the journal’s discretion for any other submissions, even if these conditions are not met.  Please find more information on the policy and a link to download a blank copy of the questionnaire here:** [**https://journals.plos.org/globalpublichealth/s/best-practices-in-research-reporting**](https://journals.plos.org/globalpublichealth/s/best-practices-in-research-reporting)**. Please upload a completed version of your questionnaire as Supporting Information when you resubmit your manuscript.**

Thank you for this instruction. We have uploaded a completed version of this questionnaire as Supporting Information (S2 Checklist).

**3. We do not publish any copyright or trademark symbols that usually accompany proprietary names, eg  ©, ®, ™  (e.g. next to drug or reagent names). Please remove all instances of trademark/copyright symbols throughout the text, including © on page 22.**

Thank you for this instruction. The symbol on page 22 has been removed and the entirety of the manuscript reviewed to confirm no other symbols are present.

**4. We have noticed that you have a list of Supporting Information legends in your manuscript. However, there are no corresponding files uploaded to the submission. Please upload them as separate files with the item type 'Supporting Information'.**

Thank you for noting this. The PLOS questionnaire on inclusivity in global research has been uploaded as a separate file with the item type ‘Supporting Information’.

Additional Editor Comments:

**Please address the comments raised by the reviewers. In particular,
- Upload a completed SRQR checklist for the reviewers and citing that in the methods
- Provide more details on the composition of the Focus Groups and the participants (e.g., number of each profession, years experience)
- Expand the implications of the findings for the study setting and the context of Tanzania**
Thank you for these comments. These have been addressed and responded to below.

**Review Comments to the Author**

**Reviewer #1: Thank you for this well written piece of Fogarty sponsored work. Paediatric surgical care in LMICs is significantly behind the care of adult surgical counterparts. Injury has also been a neglected pandemic, and thus, the authors’ work at Kilimonjaro Christian Medical Center is relevant and timely. The choice of qualitative approach is important as in-depth qualitative perspectives to barriers and facilitators to pediatric trauma care in Tanzania have not been previously explored. The rigor of the described methods and the resultant planned publication is commendable. This information might reflect the experience of several other sites in similar settings.**

Thank you so much for these kind comments about our work.

A few minor points to note:

**1. The authors need to include a checklist (Standards for Reporting Qualitative Research (SRQR)* checklist is preferred- O'Brien BC, Harris IB, Beckman TJ, Reed DA, Cook DA. Standards for reporting qualitative research: a synthesis of recommendations. Academic Medicine, Vol. 89, No. 9 / Sept 2014 DOI: 10.1097/ACM.0000000000000388) as a supporting document to benchmark their work with standards and to guide their write up. The checklist used should be mentioned in their methods.**

Thank you for this guidance. A SRQR checklist has been uploaded and mentioned in our methods as Supporting Information (S1 Checklist).

**Consider editing the topic by Standards for Reporting Qualitative Research guidelines- Identifying the study as qualitative or indicating the approach (e.g., ethnography, grounded theory) or data collection methods (e.g., interview, focus group) is recommended.**

Thank you for this suggestion. We assume you are referring to editing the title as the SRQR checklist gives these instructions for the title. We have edited our title as suggested by identifying the study as qualitative.

**Introduction- Problem formulation and Purpose or research question are well articulated.**

Thank you for this comment.

**Please clarify (Page 4, Lines 86/87)- *Due to?* the system-level challenges faced by healthcare providers in LMICs, it is important to investigate how these barriers impede quality of care for injured children. It may be best as 2 separate sentences, or even dropping the first half altogether as this has already been established in the introduction.**

Thank you for this suggestion. We have simplified the first half of the sentence to “Due to these challenges” so it is no longer repetitive with prior sentences.

**This study and all procedures were said to have received ethical approval from 2 review bodies- kindly include all ethical approval numbers in the text of the manuscript for clarity.**

Thank you for pointing out this omission. We have now included the ethical approval numbers in

the text of the manuscript for clarity on page 6, lines 118-119.

**The research team and reflexivity section appears incomplete as it only focuses on the data collectors. The authorship research team comprises 9 individuals. Consider including a deeper statement on reflexivity including the positionality of the authors/researchers including gender balance as they also interacted with the data in a variety of ways. This will further help us understand the perspectives and biases of the authors with regards to this topic.**

Thank you for this suggestion. We have included a deeper statement on reflexivity on page 9, lines 191-197: “There was no interaction between the participants and the rest of the authorship team, however the authorship team did interact with the data during analysis and manuscript preparation. Data analysis was conducted by three American researchers (two female and one male). Preliminary findings were presented and discussed with the multi-national team that included five Tanzanians (two female and three male) and six Americans (five female and one male) who brought expertise in global injuries, qualitative research, and global child health research.”

**Please insert a line in the research methods about the research paradigm (e.g., postpositivist, constructivist/ interpretivist)**

Thank you for this instruction. This has been inserted on page 5, line 116.

**Results: Please describe the composition of the Focus Groups in detail. X (y%) nurses, Z (q%) paediatric surgeons, A (b%) GPs etc. This background will help us interpret the findings appropriately.**

Thank you for this request. The only info we collected was whether or not participants were nurses or doctors, and this has been added on page 7, lines 157-158: “There were 14 (47%) doctors and 16 (53%) nurses that participated in the FGDs.”

**Table 1 can be brought up- just below the paragraph in which it was first mentioned.**

Thank you for this suggestion. Table 1 has been moved up just below the paragraph in which it

was first mentioned.

**Kindly tell us in more detail what type of practitioners responded in each quote, as these were multidisciplinary Focus Group Discussions. EMD is not detailed enough. Were these responses from the same person? Nurse 1 EMD, Doctor 1 EMD, Doctor 2 EMD etc would add sufficient detail. Same with “Paediatric Ward” and “Surgical Ward” as it would be important to know the training of these practitioners. Health practitioners were defined in the methods as physicians and nurses. The word healthcare providers is nebulous. Defining who is who is very important as perspectives of these groups can be significantly different in some working environments.**

Thank you for this request. Unfortunately we do not have this level of detail available. We performed our FGDs at the level of the ward and did not record the exact identity of each individual, so we are not able to ascertain differences in responses by provider type. Going forward, we will ensure we collect this level of data.

**Line 266-269 Pediatric-specific subspecialist care, such as pediatric neurosurgeons, pediatric orthopedic surgeons, and pediatric surgeons, are not available at KCMC. The neurosurgeons, orthopedic surgeons, and general surgeons that are able to care for these patients are often very busy given the large volume of cases in both adults and pediatrics. This statement can be strengthened by a quote. The quotation given below focuses on pediatricians.**

Thank you for this comment. We have included an additional quote specifically about the lack of pediatric specialists on page 14, lines 317-318: “*[We] mostly have general specialists available; [we] lack specialists for pediatric patients.” (Surgical Ward)*

**The final theme seems to be an amalgamation of themes, and the authors should consider the clarity of the codes- “Complexity of cases due to pre-hospital delays in patients presenting for care caused by cultural and financial barriers” focuses on pre-hospital delays (Level 2 specifically- reaching care). However, the discussion of this point centers around level 3 delays (i.e., delays in receiving care) due to financial reasons AFTER the patient has arrived at the hospital and is on the ward or in the operating room. These are distinct concepts and the codes or theme should be reconciled.**

Thank you for this request for clarification. This is a great point, and so we have changed the title of this theme from “Complexity of cases due to pre-hospital delays in patients presenting for care caused by cultural and financial barriers” to “The continuum of delays to in-hospital care caused by cultural and financial barriers.” In addition, we have made some edits to the paragraphs describing the theme to better detail how pre-existing and concurrent financial barriers for families during patient admission and cultural beliefs prior to accessing the hospital have impact on level 3 delays (i.e. delays in receiving care.) Please see page 14-15, lines 322 and 334.

**Page 19, line 402- widely developed would be preferred to widely adapted, as it comes across as if the authors are suggesting that these guidelines cannot be developed in LMIC settings for LMIC issues, and with LMIC expertise. Line 403 shows that this is not the case.**

Thank you for this correction; widely adapted has been replaced with widely developed as

suggested now in line 444.

**It is interesting that inter-professional rivalry did not arise as a theme or an issue affecting patient care negatively in these multidisciplinary focus groups as is seen in much of sub-Saharan Africa and beyond. Can the authors comment on that?**

Thank you for your comment. Yes, inter-professional rivalry was not a theme that came up during our focus group discussions and was also not specifically asked about. It is possible that this theme did not arise due to the fact that we held our FGDs with both nurse and doctor participants and thus each group might not have felt free to discuss this in the present of the other group. We have included a sentence in our limitations section acknowledging the lack of this theme in our study in the setting of previously studied inter-professional rivalry in Sub-Saharan Africa and its impact on clinical care outcomes on page 22, lines 510-514: “While a benefit, including nurses and doctors in the FGDs can also be viewed as a limitation. Previous qualitative studies in LMICs have shown inter-professional rivalry to be a theme that negatively impacts patient care,[48-50] but did not present during our study. This theme may not have presented itself because the mixed presence of nurses and doctors may have led participants to not feel free to discuss this theme.”

**Just a note of care- while it is true that learnings from KCMC can possibly be translated to other LMIC settings, the authors have consistently made a jump in the discussion to other LMICs, from the experience of a single Tertiary center in Tanzania. The authors do not deeply discuss the implications for KCMC itself, which is a potentially useful tool for implementation science. As the authors have had dissemination meetings within the hospital, can you please share some of your recommendations (possibly as an additional table, based on the barriers and facilitators identified from this qualitative work). The hospital surgical system and other East African locations can learn from your recommendations.**

Thank you for these insightful notes. Findings from the dissemination meetings were not part of our human subjects data collection and therefore cannot be added to the paper. However, the suggestions in the discussion section do pertain to KCMC, and all were discussed in the dissemination meetings as appropriate strategies to address some of these barriers that were identified in the FGDs. Thus, we have rewritten parts of the first paragraph of the discussion (page 18, lines 410-415) and the conclusion (page 23, lines 528-530) to better illustrate this: “These barriers and facilitators not only provide insight on the care of pediatric injured patients that can lead to interventions at KCMC to improve care and outcomes, but also could be translated to other tertiary referral hospitals in LMIC settings.”

**If nurses and doctors were not balanced in the focus group, then this should be included in the limitations. However, based on thematic saturation that was achieved, the authors do not justify the perceived limitation of a 30 person focus group.**

Thank you for this comment. Our focus group discussions were very nearly evenly split between nurses (53%) and doctors (47%), and this information has now been added to the methods section page 7, lines 157-158: “There were 14 (47%) doctors and 16 (53%) nurses that participated in the FGDs.” As discussed above, we think that the primary limitation was not that the FGDs were not balanced, but rather that we stratified FGDs by ward of care but not by nurses and doctors (added to the Limitations as above). We do not understand the second comment, but are happy to address it if the reviewer could please clarify what they mean.

**This is a good work and should be published.**

Thank you for this comment.

**Reviewer #2: The authors report a series of focus groups of medical providers involved in pediatric injury care in a tertiary care center in Tanzania in order to identify barriers to the provision of care along with facilitators to the provision of care. They used qualitative analysis methods to identify themes: lack of guidelines, lack of equipment, inadequate staff, and prehospital delays that were felt to impede the proper provision of care to injured children.**

Thank you for this summary.

**The study was well conducted with trained personnel conducting the groups in native language and the subsequent analysis of responses was conducted without identification of specific respondent.**

Thank you for this kind comment.

**Some questions:
1. Were there opportunities for participants to provide anonymous responses, or responses not while in the presence of other providers, potentially most important for the nurses?**

Thank you for this question. No, we did not provide an opportunity for participants to provide anonymous responses. This has been added to the Limitations section on page 21 lines 502-505 as follows: “Similar to social desirability bias, an additional limitation is that we did not provide an opportunity for participants to provide anonymous responses since data collection was in focus group discussions only. Further studies on barriers and facilitators should provide this opportunity to get more individualized responses.”

**2. What pre-hospital evaluation and transport systems are available? Are there non-cultural/financial burdens to prompt, knowledgeable prehospital care? In HIC more than half of children who die from injury do not survive to reach a hospital. Is the percentage of children who die from an injury prior to arrival to a hospital in Tanzania known?**

Thank you for these questions. There is no formal pre-hospital evaluation and transport system

such as EMS in Tanzania. The percentage of children who die from an injury prior to arrival to a

hospital in Tanzania is not currently known, but this is a very good question and is the topic of

future studies that look at pre-tertiary facility care.

**3. The study deals with care in a tertiary care center. Is that reflective of care more broadly in Tanzania? Could the authors please address that.**
Thank you for this question. No, the care at this tertiary care center is not reflective of care more

broadly in Tanzania. A sentence to address this has been added in the Limitations section lines 514-518: “A final limitation is the fact that this study was conducted at a tertiary zonal referral hospital, which is not reflective of care more broadly in Tanzania at other levels of care, such as dispensaries, health centers, district hospitals, or regional hospitals. Thus, the conclusions made in this study may only be applicable to tertiary hospitals in similar settings.”

**4. The recommended various pediatric specialists are lacking from many hospitals in the United States and seems to be very ambitious and potentially unrealistic for Tanzania. Are there other, more realistic options for LMIC? For example, very few pediatric trauma patients require a general surgical operation and could properly trained pediatricians provide sufficient care?**

Thank you for these points. We agree with you and have added the following text to page 19, lines 429-433: “There are rarely providers in LMICs with pediatric-specific training in these specialties, and expecting this to happen is ambitious and potentially unrealistic for Tanzania. A potentially more achievable solution would be to incorporate trauma management training into the medical school curriculum in LMICs in order to ensure widespread dissemination of the skills needed to manage pediatric trauma.”

**5. Are there pediatric surgeons at KCMC? If so, are they involved in the care of injured children? If not, is the level of injury care demonstrative of the level of overall pediatric care?**

Thank you for these questions. Yes, there is one surgeon at KCMC that has specialized pediatric training, and that surgeon is involved in the care of injured children when he is on-call and available. This information has been included on page 20, lines 466-468.
