# Supplementary material for: Healthcare provider perspective on barriers and facilitators in the care of pediatric injury patients at a tertiary hospital in Northern Tanzania: A qualitative study
Source: PLOS Glob Public Health. 2023 Nov 20;3(11):e0002599. doi: 10.1371/journal.pgph.0002599 (PMC10659160; doi:10.1371/journal.pgph.0002599)
Supplement: Attachment — Submitted filename: Response to Reviewers_2.docx [file pgph.0002599.s005.docx]

**Response to Reviewers:**

Journal Requirements:

**1. Please review your reference list to ensure that it is complete and correct. If you have cited papers that have been retracted, please include the rationale for doing so in the manuscript text, or remove these references and replace them with relevant current references. Any changes to the reference list should be mentioned in the rebuttal letter that accompanies your revised manuscript. If you need to cite a retracted article, indicate the article’s retracted status in the References list and also include a citation and full reference for the retraction notice.**

Thank you for this instruction. The reference list has been reviewed to ensure it is complete and correct. We are not citing any papers that have been retracted.

Additional Editor Comments:

**Thank you for addressing the comments from the reviewers. Reviewer 1 raised the following minor comments and I would appreciate if you address that and re-submit your manuscript.**

- **In response [to second reviewer] the text on page 19, lines 429-433 was edited to read “There are rarely providers in LMICs with pediatric-specific training in these specialties, '****and expecting this to happen is ambitious and potentially unrealistic for Tanzania.****'" While there is truth in difficulty of meeting this target, the terms "ambitious and potentially unrealistic" used here are strong, and can be viewed as condescending in its presentation. ... Can you kindly advise that it is removed and replaced with less strong language?**

Thank you for this comment. We agree that this text is strong and can be viewed as condescending. Thus, it has been removed. The sentences flow well without this statement, so very minor word changes were made on page 19 lines 402-403 and it now reads: “There are rarely providers in LMICs with pediatric-specific training in these specialties currently. Thus, a potentially more achievable solution would be…”
